# Trans-generational Immune Priming Protects the Eggs Only against Gram-Positive Bacteria in the Mealworm Beetle

**DOI:** 10.1371/journal.ppat.1005178

**Published:** 2015-10-02

**Authors:** Aurore Dubuffet, Caroline Zanchi, Gwendoline Boutet, Jérôme Moreau, Maria Teixeira, Yannick Moret

**Affiliations:** Équipe Écologie Évolutive, UMR CNRS 6282 BioGéoSciences, Université Bourgogne-Franche Comté, Dijon, France; Stanford University, UNITED STATES

## Abstract

In many vertebrates and invertebrates, offspring whose mothers have been exposed to pathogens can exhibit increased levels of immune activity and/or increased survival to infection. Such phenomena, called “Trans-generational immune priming” (TGIP) are expected to provide immune protection to the offspring. As the offspring and their mother may share the same environment, and consequently similar microbial threats, we expect the immune molecules present in the progeny to be specific to the microbes that immune challenged the mother. We provide evidence in the mealworm beetle *Tenebrio molitor* that the antimicrobial activity found in the eggs is only active against Gram-positive bacteria, even when females were exposed to Gram-negative bacteria or fungi. Fungi were weak inducers of TGIP while we obtained similar levels of anti-Gram-positive activity using different bacteria for the maternal challenge. Furthermore, we have identified an antibacterial peptide from the defensin family, the tenecin 1, which spectrum of activity is exclusively directed toward Gram-positive bacteria as potential contributor to this antimicrobial activity. We conclude that maternal transfer of antimicrobial activity in the eggs of *T*. *molitor* might have evolved from persistent Gram-positive bacterial pathogens between insect generations.

## Author Summary

In some insects, the immunological experience of mothers is transferred to their otherwise naïve offspring, protecting them against infection. Such a maternal effect has likely evolved from selective pressure imposed by the persistence of some microbial pathogens in the environment between insect generations. If microbes are not transmitted vertically from mother to the offspring, only those able to survive in the external environment have the highest probability to infect the offspring. Therefore, early levels of immune protection transferred by mothers to their offspring might be specific of these microbes. In this study, we found that enhanced levels of antimicrobial activity in the eggs of immune challenged females of the mealworm beetle, *Tenebrio molitor*, were only active against Gram-positive bacteria, whatever the microorganism used for the maternal challenge. Furthermore, immune challenged females with fungi rarely transferred antimicrobial activity to their eggs. The analysis of the proteins conferring antibacterial activity in the eggs of bacterially immune-challenged mothers revealed the presence of tenecin 1, an antibacterial peptide active against Gram-positive bacteria only. These results suggest that maternal transfer of antimicrobial activity in the eggs in *T*. *molitor* might have evolved from the persistence of Gram-positive bacterial pathogens between insect generations.

## Introduction

Maternal effects are of paramount importance for offspring fitness when mothers adjust the phenotype of their offspring to match the environment that they are likely to experience [1]. For instance, in many different groups of animals, the immunogenic experience of the mother is transferred to otherwise “naïve” offspring and can protect it against infection [2, 3]. Maternal transfer of immunity has been particularly well studied in vertebrates, in which infected females can transfer specific antibodies to the offspring via the placenta and milk in mammals during lactation, or via the egg yolk in birds, reptiles and fish [3, 4]. As newly born vertebrates have limited abilities to produce antibodies, this maternal transfer of immunity protects them while their own immune system becomes mature. In addition, it may further serve as signal to up-regulate the immune system of the offspring later on [3, 4].

Invertebrates lack the antibodies that vertebrate females transfer to their offspring. However, maternal transfer of immunity, also referred as trans-generational immune priming (TGIP), occurs in invertebrates too [5], suggesting that it has to be achieved by other, yet unknown, mechanisms. The effects of TGIP have been revealed through enhanced levels of immune activity and/or an increased survival to infection in primed offspring. TGIP has been found for a variety of arthropods against multiple classes of microbes and parasites [6–19]. Its trans-generational effects can be found across all life-stages of the protected progeny: from laid eggs [10, 16, 17], during the larval development [9, 11, 14, 18] and persisting even until the adult stage [12, 13,15], although its manifestation may depend on the ontogenetic stage of the offspring [18]. Furthermore, the immune protection provided to the offspring can exhibit variable levels of specificity.

Specificity measures the degree to which TGIP discriminates parasites on different levels of relatedness between the parental infection and that of the offspring. Previous studies suggest that a wide spectrum, from cross-reactive (non-specific) [6] to highly specific TGIP [7, 13] occurs in arthropods. So far specific TGIP has been demonstrated only in adult offspring and its occurrence at earlier ontogenetic stages is currently unknown. As immune defences vary with developmental stage in several insect systems [20–23], specific TGIP is also likely to vary with the ontogeny of the primed offspring. This might be particularly critical for eggs born to mothers soon after the maternal infection, as they are likely first exposed to the maternal pathogens. However the occurrence of specific TGIP in eggs is currently unknown.

In the bumblebee *Bombus terrestris* and in the mealworm beetle *Tenebrio molitor* mothers immune challenged with bacteria produce eggs containing enhanced levels of antimicrobial activity [10, 16, 17]. In insects, antimicrobial activity across all the life stages (including in eggs) generally relies on the presence of antimicrobial peptides (AMPs) and lysozymes [24–26]. Some of these molecules have a rather large spectrum of antimicrobial activity whereas others are more selective towards Gram-positive, Gram-negative or fungi [24]. Whether antimicrobial activity found into the eggs protects specifically the eggs from the pathogen that previously infected the mother is not known. However, for both bumblebees and the mealworm beetles, females and their eggs are sharing the same environment and are therefore likely exposed to the same pathogens. In this context, a specific protection in the eggs against the prevailing pathogen in the environment experienced by the mother should be particularly advantageous. Hence, within the range of specificity that AMPs and lysozymes convey to fight microbial infection, we may expect that antimicrobial activity in the eggs to be specific to the microbe that previously immune challenged the mother.

While the mechanisms through which eggs are immune protected by immune challenged mothers are currently unknown, we may propose three non-exclusive hypotheses, which support specificity of egg protection. First, immune challenged mothers may passively transfer antimicrobial peptides from their hemolymph to their eggs. Second, they may transfer mRNAs coding for immune related molecules. Under these two hypotheses, eggs should therefore exhibit a spectrum of antimicrobial activity similar to that of their mother. This might be two reasonable hypotheses since, in *T*. *molitor*, enhanced levels of antimicrobial activity are observed in eggs laid at the time where antimicrobial activity is detected in the hemolymph [17, 27]. Third, antibacterial activity found in the eggs might result from the expression of immune related molecules in the eggs themselves. Insect eggs, could be induced to produce immune effectors genes including antimicrobial peptides [25, 26, 28, 29] and it has been recently shown, in *Galleria mellonella*, that microbial immunogens that challenged mothers could be transported to ovaries and eggs where they could stimulate the expression of immune genes [29]. The production of antimicrobial factors in the eggs would differ according to the maternal challenge.

In this study, we first tested whether an immune challenge to female *T*. *molitor* affects levels of egg antimicrobial activity in a pathogen-specific manner. To this end we immune challenged females with a large range of inactivated microbial pathogens and tested the resulting antimicrobial activity of their eggs against several microorganisms, including those used to challenge mothers, using inhibition zone assays [30]. The microorganisms used were either related to one another as defined by kingdom (fungi versus bacteria), Gram type (Gram-positive versus Gram-negative) and species within the same genus. We then identified the molecular substances conferring antimicrobial activity in the eggs of bacterially immune-challenged mothers using a proteomic approach. Unexpectedly from our above hypothesis, we found that enhanced levels of antimicrobial activity in the eggs of immune challenged mothers were only active against Gram-positive bacteria but not the Gram-negative bacteria we tested, whatever the microorganism used for the maternal challenge. However, whereas maternal immune challenges with bacteria always resulted in increased levels of anti-Gram-positive activity in the eggs, no such results could be found for maternal challenges using fungi. Furthermore, the analysis of the proteins responsible for antibacterial activity in the eggs revealed the presence of tenecin 1, an antibacterial peptide known to show antibacterial activity against Gram-positive bacteria only [31]. This work suggests that pathogenic Gram-positive bacteria able to persist between generations are likely responsible of the evolution of TGIP in eggs of *T*. *molitor*.

## Results

### Bacterial-induced antibacterial activity in the eggs

We tested specificity of the maternal transfer of antimicrobial activity to the eggs by first focusing on the effects of maternal bacterial challenges on egg anti-bacterial activity. Eighteen to 23 virgin females (10 ± 1 day post emergence) per immune treatment received a single injection of a suspension of inactivated Gram-positive (*Arthrobacter globiformis* or *Bacillus thuringiensis*) or Gram-negative bacteria (*Escherichia coli* or *Serratia entomophila*) (“female treatment”) in saline solution. We chose these bacteria because they vary in their cell-wall components (DAP-type and Lys-type peptidoglycan; S1 Table, [32]) that are determinant for the expression of antimicrobial peptides (AMP) in insects [33], and because they are known to be either entomopathogenic (*Bacillus sp*., *Serratia sp*.) or ubiquitous (*A*. *globiformis*, *E*. *coli*). A group of control females was treated in the same way, but with the omission of microorganisms as a procedural control for effect of the injection (sham control mothers) and an additional group of non-injected females (naïve control mothers) was used as control. Females were then paired with a virgin and immunologically naïve male of the same age and allowed to produce eggs. Only eggs laid from day 3 to 7 post injection were collected to test their antibacterial activity because females are protecting most of their eggs at this period of time [17]. The antibacterial activity of egg extracts was tested against a range of bacteria that included those used for the maternal immune challenge (with the exception of *S*. *entomophila* replaced by *Serratia marcescens*, more often used for antimicrobial tests in laboratory) and *Bacillus subtilis* using standard zone of inhibition assays (“egg assay”) [30]. Egg antibacterial activity was first analysed by testing the probability of detecting a zone of inhibition in egg extracts for each female treatment and for each egg assay. In addition, among the egg extracts for which we detected an inhibition zone, we checked whether the diameter of this zone was different according to female treatment and egg assay.

**Fig 1:**
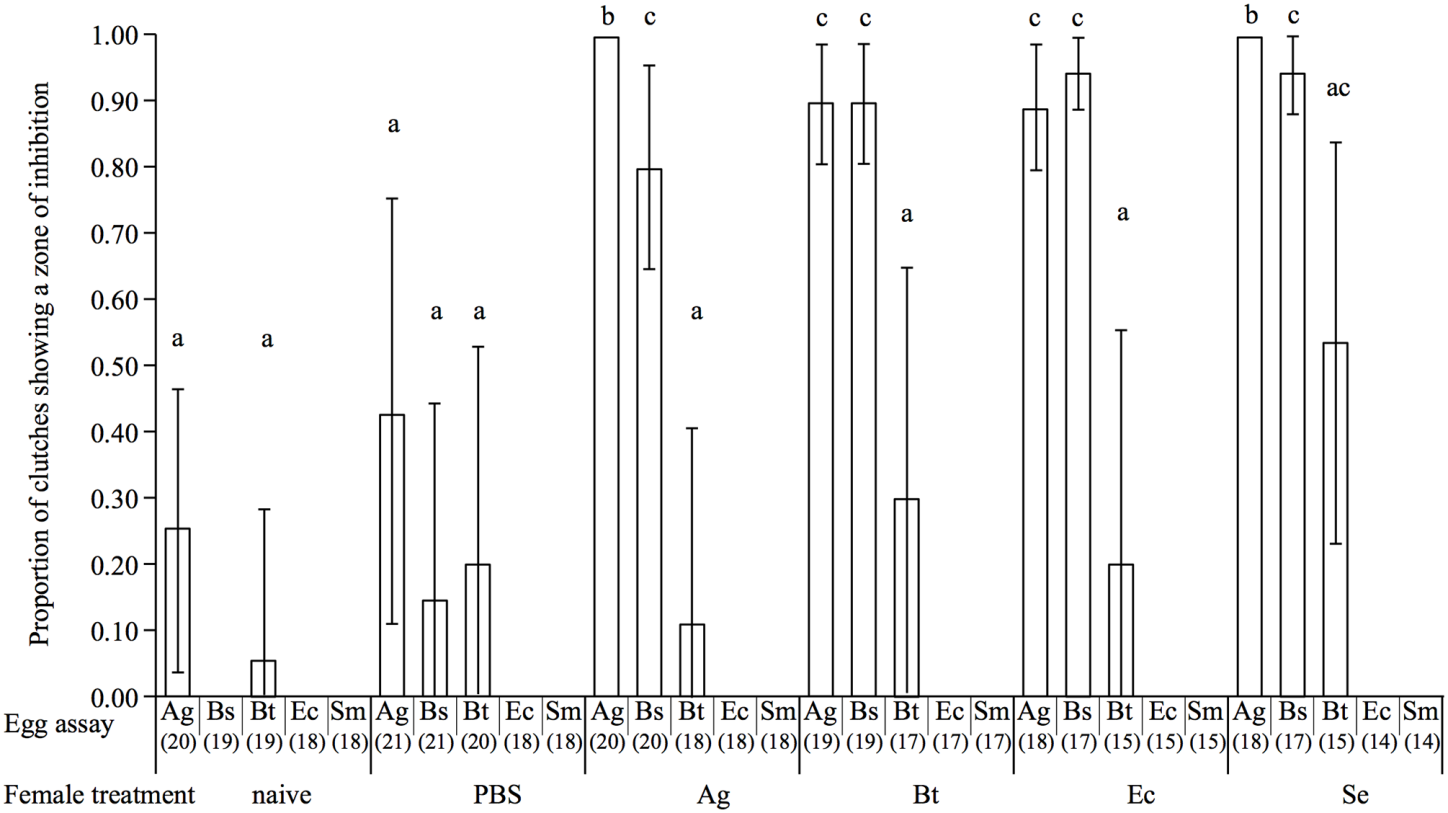
Barplot showing the mean proportion of egg extracts showing a zone of inhibition according to the microorganism on which they were tested (egg assay) and the maternal bacterial treatment. The vertical bars represent the confidence intervals at 95% (95 CI). The sample size of each treatment is stated between brackets under the egg assay. Different letters above the bars indicate significant differences according to the degree of overlap of the 95CI (p < 0.05) across female treatments. Treatments: naïve = unmanipulated females, PBS = sham-injected females, Ag = *A*. *globiformis*, Bs = *B*. *subtilis*, Bt = *B*. *thuringiensis*, Ec = *E*. *coli*, Se = *S*. *entomophila*, Sm = *S*. *marsescens*.

We found no significant interaction between the maternal treatment and the egg assay on the probability of detecting a zone of inhibition (Generalized Linear Model—GLM: female treatment * egg assay: *χ* ^2^_20, 503_ = 1.37, *p* = 0.12), suggesting that the eggs were not better protected against the specific microorganism that females had previously encountered. However, it was influenced by both the female treatment (*χ* ^2^_9, 523_ = 26.1, *p* < 0.001) and egg assay (*χ* ^2^_9, 523_ = 83.01, *p* < 0.001, Fig 1). Females challenged with bacteria produced a higher proportion of protected eggs than both naïve and sham females (see Fig 1 for corresponding Tukey's HSD test results). Concerning the egg assays, *A*. *globiformis* and *B*. *subtilis* were the bacteria that were the most susceptible to the antibacterial activity transmitted to the eggs by the challenged females, whereas the probability of detection of a zone of inhibition when the eggs were exposed to *B*. *thuringiensis* was significantly lower (see Fig 1 for corresponding Tukey's HSD test result). No zone of inhibition could be detected on *E*. *coli* and *S*. *marcescens*, while we can detect anti-Gram-negative antibacterial activity in the hemolymph of similarly immune challenged adult beetles using the same method (S1 Fig).

**Fig 2:**
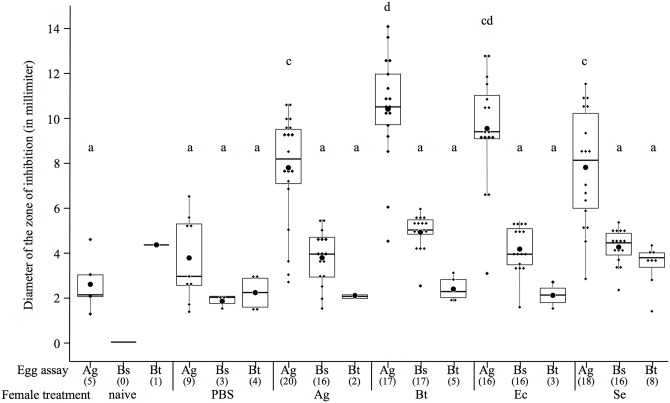
Boxplot showing the mean diameter of the zone of inhibitions (in mm) of protected egg extracts according to the microorganism on which they were tested (egg assay) and the maternal bacterial treatment. The boxes extend from the first to the third quartiles, the band inside the box represents the median. The bars outside the boxes indicate the 1.5 interquartile of the lower and upper quartiles. Each dot represents a data point, and the biggest dot represents the median. The sample size of each treatment is stated between brackets under the egg assay. Different letters above the bars indicate significant differences according to the degree of overlap of the 95CI (p < 0.05, see S2 Fig) across female treatments. Treatments: naïve = unmanipulated females, PBS = sham-injected females, Ag = *A*. *globiformis*, Bs = *B*. *subtilis*, Bt = *B*. *thuringiensis*, Ec = *E*. *coli*, Se = *S*. *entomophila*, Sm = S. *marsescens*. Were only represented, for all the maternal treatments, the clutch treatments on which a ZI could be detected.

Among the egg extracts for which we detected an inhibition zone, the size of the latter was influenced by the female treatment in interaction with the egg assay (Linear Model—LM: *F*_16, 159_ = 4.8, *p* < 0.001). However, there seems to be no support for specificity, since the egg extracts tested on *A*. *globiformis* did not produce larger zones of inhibition when they came from *A*. *globiformis*-challenged mothers than when they came from other mothers challenged with other bacteria species (Fig 2, see S2 Fig for corresponding test results). Interestingly, the egg extracts of *B*. *thuringiensis* challenged females were the ones responsible for the biggest zone of inhibition on *A*. *globiformis* (Fig 2). In the case of the egg extracts tested on *B*. *subtilis* and *B*. *thuringiensis*, the size of the zone of inhibition they produced was similar between female bacterial treatments, and were not significantly different compared to naïve and PBS-challenged females (Fig 2).

Thus all the bacteria species we used to immune challenge mothers induced enhanced levels of antibacterial activity in the eggs. However, whatever the type of bacteria (Gram-negative or Gram-positive) that challenged the mothers, antimicrobial activity found in the eggs was only active against Gram-positive bacteria.

### Fungal-induced challenge antimicrobial activity in the eggs

Test of specificity of the maternal transfer of antimicrobial activity to the eggs was further extended to fungal maternal challenges using the same method as above. Females (22 to 23 per immune treatment group) were immune challenged with killed yeasts of *Candida albicans*, or spores of the entomopahogenic fungus, *Metarhizium anisopliae*. Immune challenged females with killed *A*. *globiformis* and sham injected ones were also included in this experiment as positive and negative controls, respectively. The resulting egg extracts were tested against yeasts (*C*. *albicans*), fungi (*M*. *anisopliae*), Gram-positive bacteria (*A*. *globiformis* and *B*. *subtilis*) and Gram-negative bacteria (*E*. *coli*, *S*. *marcescens*), using standard zone inhibition assays.

**Fig 3:**
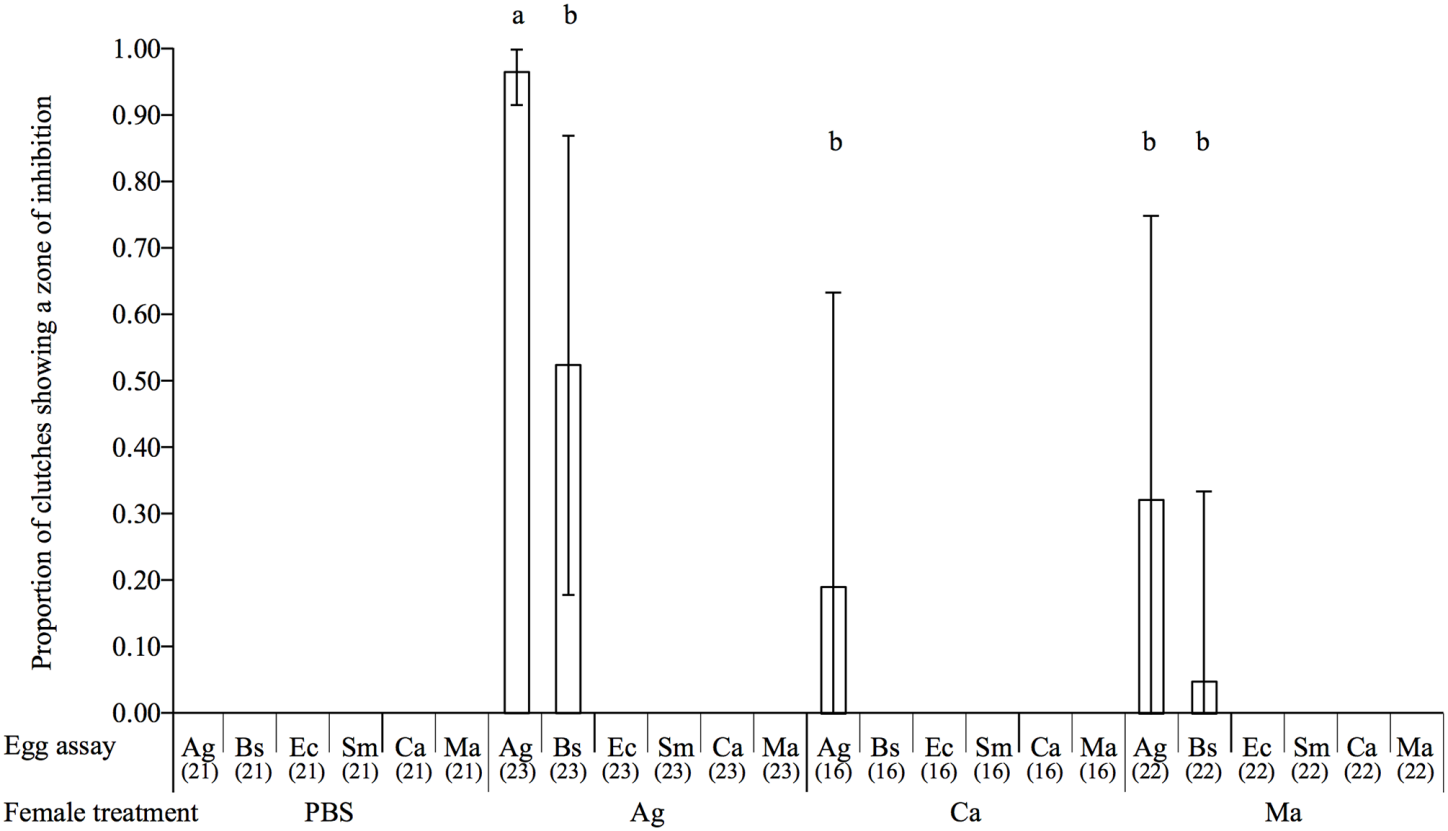
Barplot showing the mean proportion of egg extracts showing a zone of inhibition according to the microorganism on which they were tested (egg assay) and the maternal microbial treatment. The vertical bars represent the confidence intervals at 95% (95CI). The sample size of each treatment is stated between brackets under the egg assay. Different letters above the bars indicate significant differences according to the degree of overlap of the 95CI (p < 0.05) across female treatments. Treatments: PBS = sham-injected females, Ag = *A*. *globiformis*, Bs = *B*. *subtilis*, Ec = *E*. *coli*, Sm = *S*. *marcescens*, Ca = *C*. *albicans*, Ma = *M*. *anisopliae*.

As in the previous experiment, we found no significant interaction between the maternal treatment and the egg assay on the probability of detecting a zone of inhibition (GLM: female treatment * egg assay: *χ* ^2^_28, 348_ = 0.07, *p* = 1), but the female treatment (*χ* ^2^_10, 358_ = 27.9, *p* < 0.001) and the egg assay (*χ* ^2^_10, 358_ = 29.7, *p* < 0.001) had a significant effect. *A*. *globiformis*-challenged females produced a higher proportion of protected eggs compared to *C*. *albicans* and *M*. *anisopliae*-challenged females (Fig 3). Similarly to our previous experiment, there was no evidence of specificity since an antibacterial activity could only be detected against *A*. *globiformis* and *B*. *subtilis*, and never on *E*. *coli*, *S*. *marcescens*, *C*. *albicans* and *M*. *anisopliae* (Fig 3). In addition, contrary to *A*. *globiformis*-challenged females, eggs of *C*. *albicans*-challenged, females did not produce any zone of inhibition on *B*. *subtilis*, and, among the eggs of the *M*. *anisopliae*-challenged females only those of a single female produced a zone of inhibition (see Fig 3 for corresponding test results).

**Fig 4:**
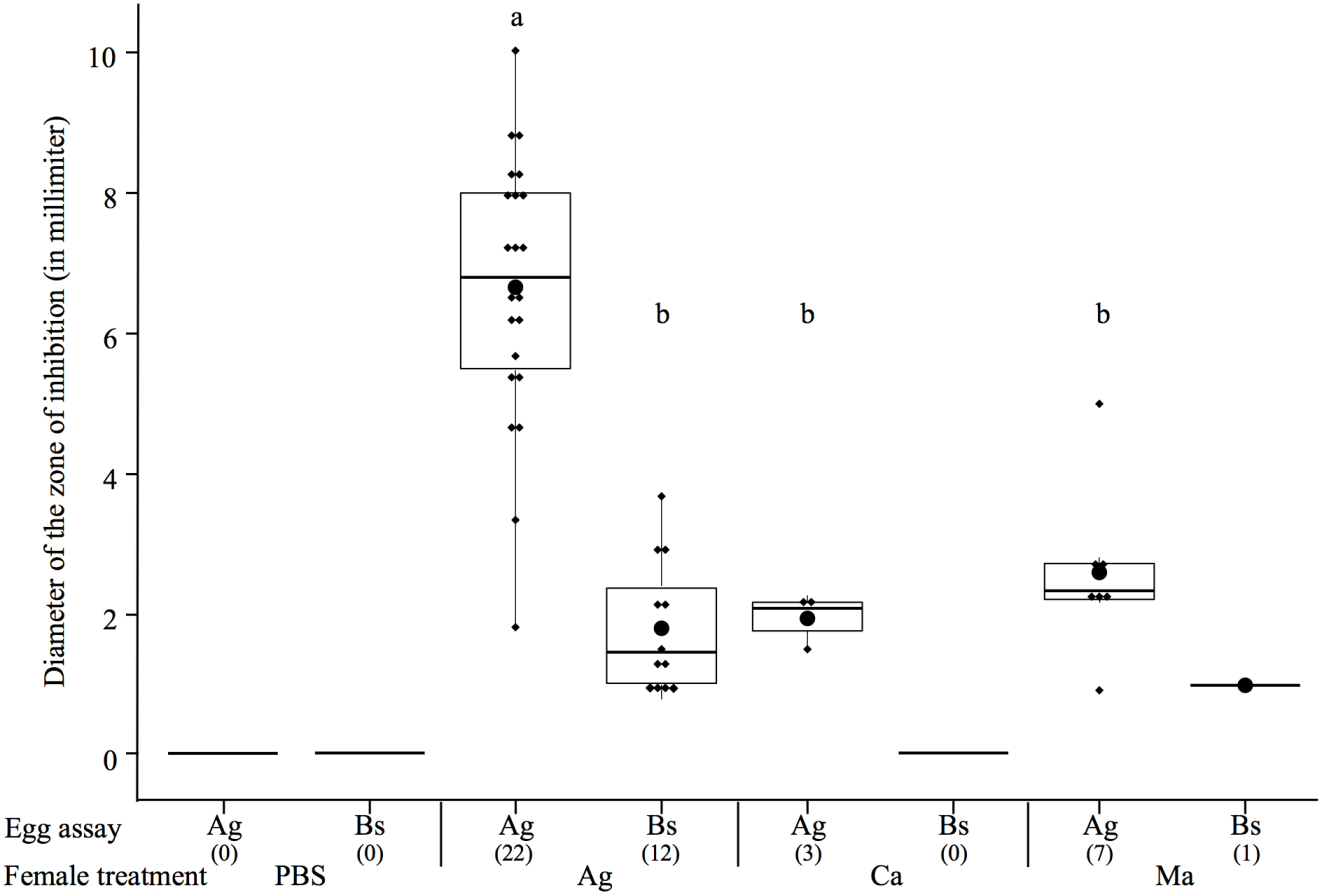
Boxplot showing the mean diameter of the zone of inhibitions (in mm) of protected egg extracts according to the microorganism on which they were tested (egg assay) and the maternal microbial treatment. The boxes extend from the first to the third quartiles, the band inside the box represents the median. The bars outside the boxes indicate the 1.5 interquartile of the lower and upper quartiles. Each dot represents a data point, and the biggest dot represents the median. The sample size of each treatment is stated between brackets under the egg assay. Different letters above the bars indicate significant differences according to the degree of overlap of the 95CI (p < 0.05, see S3 Fig) across female treatments. Treatments: PBS = sham-injected females, Ag = *A*. *globiformis*, Bs = *B*. *subtilis*, Ca = *C*. *albicans*, Ma = *M*. *anisopliae*. Were only represented, for all the maternal treatments, the clutch treatments on which a ZI could be detected.

There was also no effect of the female treatment in interaction with the egg assay on the size of zone of inhibition produced by the eggs among the clutches that exhibited antimicrobial activity (LM: *F*_4, 40_ = 3.37, *p* = 0.07). However, both female treatment (*F*_3, 41_ = 22.59, *p* < 0.001) and egg assay (*F*_3, 41_ = 67.85, *p* < 0.001) had a significant effect (Fig 4). Females challenged with *A*. *globiformis* produced the eggs with the highest antibacterial activity compared to females challenged with *C*. *albicans* and *M*. *anisopliae* (Fig 4). Consistently with the previous experiment, *A*. *globiformis*-challenged females produced eggs with a larger zone of inhibition on *A*. *globiformis* compared to *B*. *subtilis* (Fig 4, see S3 Fig for corresponding test results).

These results show that maternal challenges with fungi rarely led to the presence of antimicrobial activity in the eggs than maternal challenge with bacteria. However, when they do, antimicrobial activity found in the eggs is restricted toward Gram-positive bacteria, as for the bacterial immune challenges.

### Identification of egg antibacterial substances

Based on above results, we aimed to identify the substance(s) responsible for egg antibacterial activity from eggs produced by bacterially immune challenged mothers. To this purpose, we used egg extracts from immune challenged females with killed *A*. *globiformis* (N = 8), *B*. *thuringiensis* (N = 16), *E*. *coli* (N = 13) or *S*. *entomophila* (N = 11) and those from sham-injected (N = 21) and naïve (N = 7) control females.

**Fig 5:**
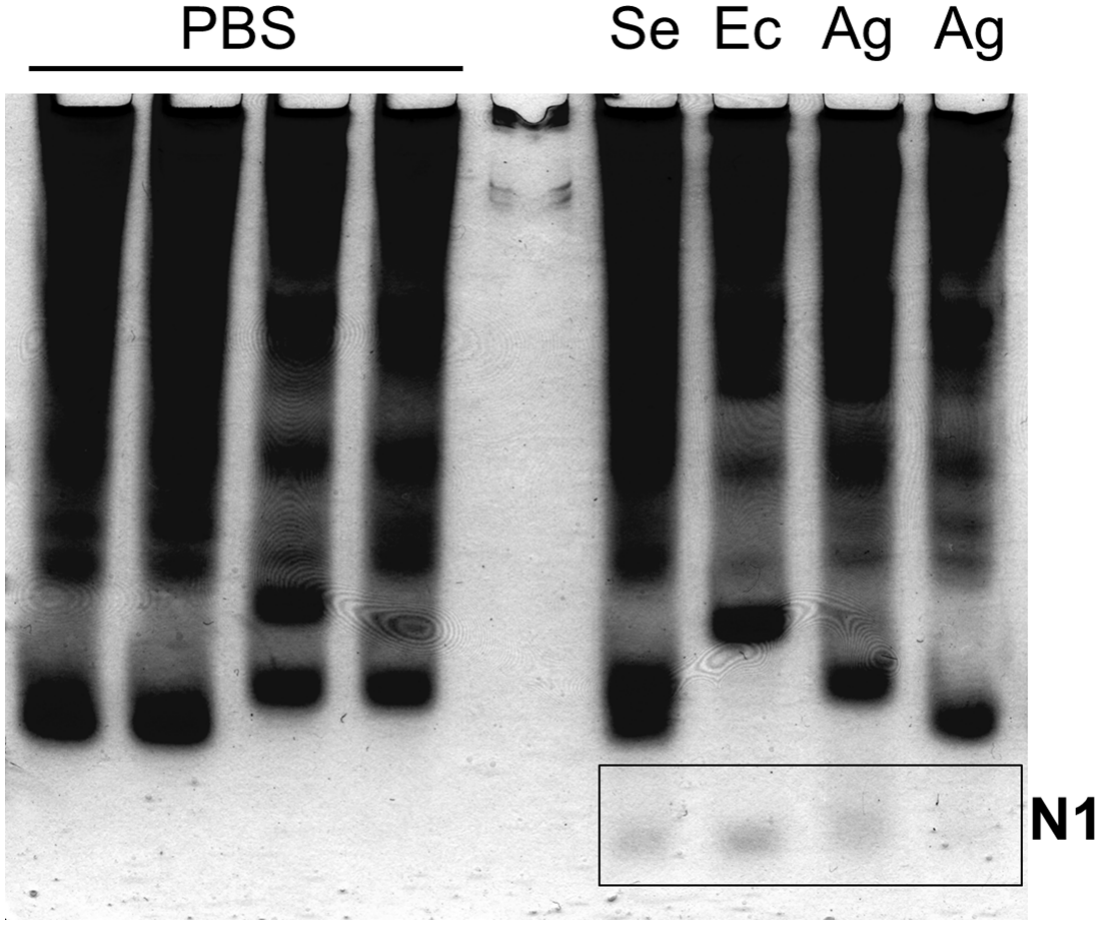
Detection of the N1 band in egg extracts from females injected with bacteria. Protein profiles of eggs from females injected with inactivated bacteria (Se: *S*. *entomophila*, Ec: *E*. *coli*, Ag: *A*. *globiformis*) and eggs from sham control females (injected with PBS), resolved on an AU-PAGE gel. The N1 band, only detected in egg extracts from bacteria-injected females, is shown in the rectangle.

Egg extracts exposed to proteinase K lost their antibacterial activity on *A*. *globiformis* test plates (S4 Fig), confirming the proteinaceous nature of the egg antibacterial compounds. Then, in order to determine whether the maternal treatment influenced the eggs proteome, we compared the protein profile of the above egg extracts using Acid Urea—Polyacryamide Gel Electrophoresis (AU-PAGE) [34] and Tricine-SDS PAGE [35]. The protein profiles produced by AU-PAGE revealed the presence of an additional band of protein(s) (N1) in all egg extracts of immune challenged mothers with *B*. *thuringiensis*, *E*. *coli* and *S*. *entomophila*, in 88% of eggs from mothers immune challenged with *A*. *globiformis*, and never in eggs of sham injected and naïve control mothers (Fig 5). Similar results were obtained using Tricine-SDS PAGE analysis (S5 Fig).

Protein band(s) containing the protein(s) responsible for antibacterial activity in the eggs were then localized using a Gel Overlay Assay [34]. This assay consisted in transferring an AU-PAGE gel after electrophoresis onto a zone of inhibition test plate seeded with *A*. *globiformis* to test the antibacterial activity of each protein band. The assay revealed the presence of a single zone of bacterial growth inhibition corresponding to the N1 band in the colloidal blue-stained gel run in parallel. This assay revealed that the N1 band contained proteins with anti-Gram positive activity (S6 Fig). To get better resolution on the size of the protein band, a slice of AU-PAGE gel from the antibacterial region was electrophoresed into a Tricine SDS-PAGE gel, and resolved into a single band of about 5 kDa, which is similar to the molecular mass of D1 band proteins (S5 Fig).

Two protein bands corresponding to N1 on AU-PAGE gels after migration of egg extracts from *B*. *thuringiensis* and *E*. *coli*–injected females, and one corresponding to D1 on Tricine SDS-PAGE gels obtained after migration of egg extracts from *S*. *entomophila*–injected female (S5 Fig) were analysed by mass-spectrometry (LC-MS/MS) for protein identification. This analysis revealed the presence of tenecin-1 in both the N1 and D1 bands, whatever the bacterial maternal treatment (Table 1). Tenecin-1 is a 4.5 kDa antimicrobial peptide from the defensing family inhibiting the growth of Gram-positive bacteria [31].

**Table 1:** Results of mass spectrometry analysis. Identification of proteins contained in the protein bands specifically found in egg extracts from bacteria-challenged females (N1: from native AU-PAGE gel, D1: from Tricine-SDS-gel).

Egg extracts collected from females injected with:	Protein band	Peptide sequences recovered	Representative coding sequences (UniProtKB/Swiss-Prot) (% coverage)	ID with significant BLAST similarity
*B*. *thuringiensis*	N1	VTCDILSVEAK, GVKLNDAACAAHCLFR, LNDAACAAHCLFR, GRSGGYCNGKR	Q27023 (51.2%)	*Tenebrio molitor* Tenecin-1
*E*. *coli*	N1	VTCDILSVEAK	Q27023 (13.1%)	*Tenebrio molitor* Tenecin-1
*S*. *entomophila*	D1	VTCDILSVEAK, LSVEAK, LNDAACAAHCLFR	Q27023 (28.6%)	*Tenebrio molitor* Tenecin-1

## Discussion

We report three novel findings about maternal transfer of immunity in the eggs of the mealworm beetle *T*. *molitor* following a microbial immune challenge of the mothers. First, we found that levels of antimicrobial activity in eggs resulting from maternal challenge differed according to the kingdom of the microbes used for the maternal immune challenge. Whereas the bacteria species we used to immune challenge mothers induced enhanced levels of antimicrobial activity in the eggs (Fig 1), the use of fungi rarely did (Fig 3). Second, whatever the type of the microbes that challenged the mothers (fungi, Gram-negative or Gram-positive bacteria), antimicrobial substances found in the eggs were only active against Gram-positive bacteria. Third, we provide reasonable evidence that antibacterial activity directed toward Gram-positive bacteria in eggs of bacterially immune-challenged mothers is caused by the presence of tenecin 1, an antimicrobial peptide for which no other function than an activity against Gram+ bacteria has been reported [31]. Hence, antimicrobial activity in *T*. *molitor* eggs is mainly induced when mothers are challenged by bacteria and targets Gram-positive bacteria.

At first glance, our results contrast with those of a previous study, which found that the maternal immune protection of the offspring of the red flour beetle, *Tribolium castaneum*, was pathogen specific [13]. In this species, survival was increased in the adult offspring when the bacteria strain used on the progeny was the same than the one used in the maternal challenge. This would mean either that the degree of specificity of TGIP can differ between even closely related species, or that the immune protection of the offspring becomes pathogen specific at a later life stage. The effects of TGIP indeed differ across life stages [18]. In *T*. *molitor* for example, TGIP results in elevated levels of antimicrobial activity in eggs and larvae, but not in adults where increased haemocyte counts were observed [9, 15, 16]. Such a qualitative variation in the expression of enhanced immunity across life stages of the offspring may have several putative causes. For instance, it is possible that physiological constrains during the ontogeny of the developing offspring restrain the involvement of some immune effectors at certain life stages. Alternatively, ecological differences between eggs, newborn larvae and adults may differentially expose the offspring to microbes present in the environment. As a consequence, the immune effectors involved in the protection of the offspring at each life-stage might be optimal. Further investigations are needed to test these hypotheses.

It is currently unknown whether antibacterial activity in *T*. *molitor* eggs originates from the mothers or from the laid eggs themselves, although both origins are not exclusive. In various vertebrate and invertebrate species, maternally expressed immune factors are transferred into or onto the offspring, even in absence of any experimental maternal challenge [36–38]. As enhanced levels of antimicrobial activity are observed in eggs laid at the time where antimicrobial activity is detected in the hemolymph of *T*. *molitor* females [17, 27], one could reasonably hypothesize that the antimicrobial activity in the eggs originates from the antimicrobial peptide passively transferred by females to the eggs. In that case, its spectrum of activity would be a reflection of the local disease environment and should mirror that of the female’s hemolymph. In *T*. *molitor*, exposure to any kind of bacterial peptidoglycan (Lys-type or DAP-type) induces a strong anti-Gram-positive activity in the hemolymph of nymphs and adults ([27, 39] and S1 Fig). Similarly, exposure to DAP-type peptidoglycans (carried by Gram-negative and *Bacillus* bacteria) induces anti-Gram-negative activity ([39] and S1 Fig). If immune challenged females passively transfer antimicrobial substances in their eggs and assuming that all of them are equally transferred in eggs, then both anti-Gram-positive and anti-Gram-negative activity should be found in eggs of challenged females. In this study, anti-Gram-positive activity was found in eggs of bacterially challenged females, whatever the bacterial challenge. However, we failed to detect anti-Gram-negative activity in the eggs of females challenged with Gram-negative or *Bacillus* bacteria while we can detect it in the hemolymph of females using the same method (S1 Fig). While this would argue against a passive transfer of antibacterial peptides to the eggs by mothers, it should be noted that anti-Gram-negative activity in the hemolymph of mothers is much weaker than the anti-Gram-positive one (S1 Fig). This could result in very low or undetectable anti-Gram-negative activity in the eggs if mothers passively transfer antimicrobial activity to their eggs. Therefore, we cannot rule out the possibility that mothers can passively transfer antibacterial peptides in the eggs. Alternatively, antibacterial activity found in the eggs might be produced in the eggs themselves, in which the expression of genes encoding antimicrobial factors would differ according to the occurrence of a maternal challenge. Insect eggs, including coleopteran species, are able to express immune effectors genes such as antimicrobial peptides [25, 26, 28, 29]. In *G*. *mellonela*, immune gene expression in eggs was recently proposed to be induced by the depostion of the immunogens that challenged mothers in the eggs, which potentially support the expression of specific transgenerational immune responses [29]. If *T*. *molitor* eggs express genes coding for antimicrobial factors, they may thus exclusively express tenecin 1, suggesting that egg response to immunogens differs from that of their mother. Additional analyses found that transcripts of tenecin-1 were found to be abundant in eggs of immune-challenged females compare to those of control females (S1 Text). While it suggests that the peptides can be produced in the eggs, it does not rule out the possibility that transcripts can be provided by the mothers. Further studies will determine whether *T*. *molitor* eggs can express genes encoding antimicrobial factors, such as lysozymes and antimicrobial peptides, and whether such expression differ according to the maternal treatment. Our results might also be explained in a context of “microbiota effect”, when resident microbes replicate before being vertically transmitted upon infection of the host. Heritable bacteria are widespread in insects [40] and could persist at an undetectable concentration to the host immune system. Here, one might imagine that *T*. *molitor* females could harbour Gram-positive bacteria that are triggered into vertical transmission upon infection by any bacterial species, explaining the presence of antibacterial activity directed toward Gram-positive bacteria in eggs of immune challenge females. However, to our knowledge, *T*. *molitor* has yet never been reported to house covert bacterial infection or symbiotic bacteria [41]. Furthermore, we found no convincing indications of the presence of covert bacterial infection in ovaries and eggs of both control and immune challenged *T*. *molitor* females (S2 Text).

From an ecological and evolutionary point of view, the pattern of antimicrobial protection of the eggs may result from selective pressures imposed by the dominant microbial threat of the mealworm beetle. Insect eggs are known to suffer from microbial infection [42, 43] and our results suggest that Gram-positive bacteria might be more threatening than any other microbes in the natural environment of *T*. *molitor*. Microbial pathogens of *T*. *molitor* are currently barely known. To our knowledge, only three bacterial species have been described so far as pathogens of *T*. *molitor* and all are Gram-positive bacteria: *Bacillus cereus*, *Bacillus thuringiensis* and *Brevibacillus laterosporus* [44]. Other might be discovered in the future. Further studies will determine whether these or other Gram-positive bacteria are indeed the most abundant or threatening parasites for *T*. *molitor*, whether they can infest *T*. *molitor* eggs and whether antimicrobial activity provisioned in the eggs is beneficial against these microbes.

Even if Gram-positive bacteria are not the most abundant, they are probably the group of bacteria that are the most able to persist in the external environment by forming endospores. Endospore formation is limited to several genera of Gram-positive bacteria that include entomopathogenic bacteria such as *Bacillus* and *Brevibacillus* [45]. In the context of persisting diseases across generations, we may expect that when microbes are not transmitted vertically from mother to the offspring, only those able to survive in the external environment, such as endospore forming bacteria, have the highest probability to infect the offspring. Therefore, early levels of immune protection transferred by mothers to their offspring might be specific of these microbes. However, because fungi also form persistent spores, the above argument should hold for them too, especially knowing that insect eggs can suffer from fungi infections [42]. In contrast, our results show that fungi were weak inducers of TGIP in the eggs of *T*. *molitor*. One might hypothesise that immune effectors mobilized against fungi could not be transferred to the eggs or that females may use other maternal effects to avoid the infection of their eggs by entomopathogenic fungus [46].

To summarize, we found that maternal transfer of antimicrobial activity in eggs of *T*. *molitor* is mainly induced by bacterial immune challenges. However, whatever the microbial pathogen that challenged the mothers, antimicrobial substances transferred to the eggs were only active against Gram-positive bacteria because of the presence of tenecin 1. Our results suggest that this antimicrobial peptide found in the eggs is unlikely to originate from the female’s hemolymph. To our knowledge, our study is the first to provide a substantial characterization of the molecular mechanism involved in TGIP in an insect. Besides, we propose that maternal transfer of antimicrobial activity in the eggs of *T*. *molitor* might have evolved from the persistence of Gram-positive bacterial pathogens between insect generations. Detailed characterization of the community of pathogens associated with *T*. *molitor* is required to test this hypothesis.

## Materials and Methods

### Insect cultures and experimental designs

Mealworm beetles originated from an outbred stock culture maintained in our laboratory in bran flour added with *ad libitum* access to water and regularly added with proteins (piglet flour), apple and bread. Pupae were then collected from these stock cultures and adults were maintained individually after emergence in a Petri dish supplied with bran flour a piece of apple and water for ten days. The experiments used virgin adult beetles of controlled age (10 ± 1 day post emergence). Due to practical limitations, the study was conducted in two successive experiments. The first experiment focused mainly on the effects of maternal bacterial challenges on the eggs anti-bacterial activity whereas the second experiment mainly focussed on the effects of maternal fungal immune challenges. Females were immune challenged (“female treatment”) by injection of a 5-μL suspension of inactivated microorganisms in phosphate buffer saline (PBS 10 mM, pH 7.4) after being chilled on ice for 10 min. Control females were treated in the same way, but with the omission of microorganisms as a procedural control for effect of the injection (sham control mothers). In the first experiment, an additional group of non-injected females (naïve control mothers) was used as control. Only sham control mothers were used in the second experiment because the first experiment reveals that in antimicrobial activity in eggs of sham control mothers was similar to that of naïve control mothers. Immediately after their immune treatment, the females were paired with a virgin and immunologically naïve male of the same age and allowed to produce eggs in a Petri dish supplied with wheat flour, apple and water in standard laboratory conditions (25°C, 70% RH; dark). Egg were collected every second day from day 3 to day 7 post injection because females are protecting most of their eggs at this period of time [17]. Two samples of 10 to 12 eggs per female were collected at random during this period of time to prepare egg extracts which antimicrobial activity was tested against the above microorganisms using inhibition zone assays [30].

### Preparation of the microorganisms used for injection of mothers

Bacteria and yeasts were grown overnight at 28°C in LB and Sabouraud liquid respectively. *M*. *anisopliae* was cultured on Sabouraud-agar plates for four days at 28°C. Microorganisms were then inactivated in 0.5% formaldehyde prepared in PBS for 30 minutes, rinsed three times in PBS, and their concentration adjusted to 10^8^ microorganisms per ml using a Neubauer improved cell counting chamber. Aliquots were kept at -20°C until use.

### Preparation of egg extracts and inhibition zone assays

Egg extracts were prepared by smashing eggs into an acetic acid solution (0.05%, 5 μl per egg) followed by a 2 min centrifugation at 3600 rpm. Supernatants were divided into six to seven aliquots and kept at -20°C until antibacterial tests could be carried out.

Antimicrobial activity in egg extracts was measured using standard zone of inhibition assays [30]. Microorganisms were cultured as described above and seeded to 1% agar plates at a final concentration of 10^5^ microorganisms / ml (LB-agar plates for bacteria, Sabouraud-agar plates for *C*. *albicans* and *M*. *anisopliae*). Six millilitres of seeded medium was poured into a Petri dish and sample wells were then made using a Pasteur pipette fitted with a ball pump. Two microliters of egg extract was added to each well. Plates were then incubated at 28°C for 24 or 48 h according to the tested microorganism after which the diameter of inhibition zones was measured. Antimicrobial activity of egg extracts obtained from the two random samples of eggs (see above) produced by each female was tested in triplicate for each microorganism (egg assay) and the mean of these 6 values was used as data point.

### Protein profiles of egg extracts from bacterially immune challenged mothers

The proteinaceous nature of the antimicrobial compounds in egg extracts of bacterially immune challenged mothers was assessed by testing the persistence of their antibacterial activity against *A*. *globiformis* after being incubated with proteinase K (200 μg/mL) for 2 hours at 37°C. The influence of maternal immune challenge on the proteome of their eggs was examined by comparing the protein profile from egg extracts (obtained each from 5 eggs) produced by immune challenged mothers with *A*. *globiformis*, *B*. *thuringiensis*, *E*. *coli*, *S*. *entomophila* with that of sham control and naïve control mothers using Acid Urea—Polyacryamide Gel Electrophoresis (AU-PAGE) [34] and Tricine-SDS PAGE [35]. For the AU-PAGE analysis, extracts were mixed in loading buffer (9M urea in 5% acetic acid with methyl green as tracking dye) and loaded on a 15% AU-PAGE, which was pre-run at reversed polarity for 1h at 150 V using 5% acetic acid as running buffer. After 100 min of migration at 150V at reversed polarity, the gels were stained using a colloidal blue solution [47]. Tricine-SDS PAGE gels consisted in a 16.5% separating and an 8% stacking gels. After a 140 minutes run at 90V, the gels were stained using colloidal blue. The antimicrobial activity of all the extracts we used was assessed in parallel using zone of inhibition assays (see above).

### Gel Overlay Assay

Protein band(s) responsible for antibacterial activity in the eggs of bacterially immune challenged mothers were localized using a Gel Overlay Assay [34]. Egg extracts (10 eggs diluted in 25 μl of 0.05% acetic acid) were subjected to non-denaturing AU-PAGE in duplicate, as described above (except that loading buffer did not contain methyl green). The gel was cut into two identical halves. One half was rinsed twice for 10 min with 10 mM Sodium Phosphate Buffer pH 7.4 to remove excess of acetic acid and urea. The rinsed gel was then placed on LB-agar Petri dish prepared with low-electroendosmosis-type agarose containing *A*. *globiformis* (10^5^ bacteria per ml). After incubation for 3h allowing transfer of the electrophoresed polypeptides to the underlying agar plate, the electrophoresis gel was removed and the Petri dish was incubated for 36 hours at 28°C to allow bacterial growth. Zones into which antimicrobial proteins or peptides had diffused were identified as bacterial colony-free regions. The second half of the gel was stained using colloidal blue solution.

### Mass spectrometry

Protein bands of interest from egg extracts produced by immune challenged females with *B*. *thuringiensis*, *E*. *coli* and *S*. *entomophila* were analysed by mass-spectrometry (LC-MS/MS). Native proteins (N1 band) and denatured ones (D1) were excised from colloidal blue stained AU-Page and Tris-SDS gels, respectively. Gel pieces were distained (NH_4_CO_3_ 50mM/CH_3_CN 50/50) and digested with trypsin (0.3 μg/digestion) at 30°C for 12 hours. Peptide sequences were determined by mass spectrometry performed using a LTQ Velos instrument (Dual Pressure Linear Ion Trap) equipped with a nanospray source (ThermoFisher Scientific) and coupled to a U3000 nanoLC system (ThermoFisher Scientific). Proteins identification was performed with the MASCOT Algorithm from the Proteome Discoverer software v1.1 (ThermoFisher Scientific) against the UniProtKB/Swiss-Prot database (Dec 2012).

### Statistics

Specificity of the transgenerational immune response was analysed by first testing the probability of detecting a zone of inhibition in egg extracts for each female treatment and for each egg assay. Among the egg extracts for which we detected an inhibition zone, we checked whether the diameter of this zone was different according to female treatment and egg assay. The probability of detecting a zone of inhibition according to the female treatment and the egg assay was first analysed using a Generalized Linear Model (GLM) fitted with a binomial distribution (presence/absence of a zone of inhibition produced by a clutch for a given microorganism). Then the size of the zone of inhibition of the protected egg extracts according to the female treatment and egg assay was analysed with a Linear Model (LM). Egg extracts from the same female were assessed on different microorganisms, causing pseudoreplication and ideally the above analyses should have included the female ID as a random factor in a Generalized Linear Mixed Model (GLMM) and a Linear Mixed Model (LMM). However, the integration of a random factor in these models leads to their overparameterization, and the impossibility to test for the interaction between female and egg treatments. Since the results of the regular or mixed-effect models including the simple effects without interactions were consistent, we decided to continue our analyses with a simple GLM and a LM on these response variables. For all analyses, we checked if the residuals of the models were normally distributed and their variance homogeneous, in order to confirm the choice of a given distribution and explanatory variables. We performed backwards-stepwise regression as a mean for model simplification. We started by including both female treatment and egg assay and their interaction, and proceeded to remove non-significant terms. Comparisons of fits of the different models are provided in S3 Text. Differences between each female treatment and egg assay were highlighted by estimating the degree of overlap of the 95% CI, following the recommendations of [48]. The difference was considered significant when the 95% CI did not overlap on more than half of their length (p<0.05) [49]. The confidence intervals are represented on the bar plots of Figs 1 and 3. For Figs 2 and 4, they are represented with the estimates of the models (package ggplot2) in S2 and S3 Figs, respectively. All the data were analysed using R software [49]. The GLMM and LMM were performed with le lme4 package [50]. All data files are available from the Dryad database at doi:10.5061/dryad.4g40g. [51].

### Accession number

The accession number for the *T*. *molitor* antimicrobial peptide tenecin 1 is Q27023.

## Supporting Information

S1 Table Microorganisms used in this study. (DOCX)

S1 Fig Boxplot showing the diameter of the zone of inhibitions (in mm) obtained using hemolymph of females according to their immune treatment. Treatments: PBS = sham-injected females, Ag = *A*. *globiformis*, Bt = *B*. *thuringiensis*, Ec = *E*. *coli*. a) hemolymph tested on *A*. *globiformis*, b) hemolymph tested on *E*. *coli*. The left panel (a) shows the anti-*A*. *globiformis* activity and the right panel (b) shows the anti-*E*. *coli* activity in the hemolymph of female beetles according to their immune treatment. Sample size of each treatment is stated under the female treatment (n = x). Females were injected using the same procedure as described in the Materials and Method section. Six μL of hemolymph per female was collected and diluted in 24 μL of PBS supplemented with PTU. For each female, 3 replicates of 2 μL were tested using inhibition zone assay, as described in the Materials and Method section. The edges of the rectangles represent the first and the third quartiles, the central features are the medians, the dashed lines are the maxima and minima.
(DOCX)

S2 Fig Plot of the estimates (dots) and 95% CI (bars) of the LM carried on protected egg extracts according to the microorganisms on which they were tested (egg assay) and the maternal immune treatment. The sample size of each treatment is stated under the egg assay (n = x). Treatments: naïve = unmanipulated females, PBS = Sham injected females, Ag = *A*. *globiformis*, Bs = B. *subtilis*, Bt = *B*. *thuringiensis*, Ec = *E*. *coli*, Se = *S*. *entomophila*.
(TIF)

S3 Fig Plot of the estimates (dots) and 95% CI (bars) of the LM carried on protected egg extracts according to the microorganisms on which they were tested (egg assay) and the maternal immune treatment. The sample size of each treatment is stated under the egg assay (n = x). Treatments: PBS = Sham injected females, Ag = *A*. *globiformis*, Bs = *B*. *subtilis*, Ca = *C*. *albicans*, Ma = *M*. *anisopliae*.
(TIF)

S4 Fig Antibacterial activity of egg extracts from immune-challenged females of *Tenebrio molitor* treated with proteinase K. Antibacterial activity tested against *Arthrobacter globiformis* of egg extracts from control (PBS, right column) and immune-challenged females with *Bacillus thuringiensis* (Bt, left column) either non-treated (Bef.), incubated with proteinase K for 2 hours at 37°C (PK) or incubated without proteinase K for 2 hours at 37°C (S/S). Treatment with proteinase K inhibited the antibacterial activity of the egg extract from Bt-immune-challenged females revealing the proteinaceous nature of the antimicrobial compounds in these egg extracts.
(DOCX)

S5 Fig Detection of the D1 band in egg extracts from females injected with bacteria. Protein profiles of eggs from females injected either with PBS or with *B*. *thuringiensis* (Bt) are compared using a Tricine-SDS PAGE gel. The D1 band, only detected in egg extracts from Bt-injected females, is shown in the rectangle. In total, we detected the D1 band in 11 of the 14 protein profiles of egg extracts from Bt-injected females, but never in the 5 egg extracts from sham-injected control females.
(DOCX)

S6 Fig Localization of the antimicrobial proteins by Gel Overlay Assay. Egg extracts from PBS-injected and *S*. *entomophila*-injected females were run in duplicate sets on a single native AU-PAGE gel. One half of the gel was stained with colloidal blue (left), the other was used on a Gel Overlay Assay with *A*. *globiformis* (right). A zone of bacterial growth inhibition (arrowhead) is observed at the level of the N1 band (arrow), only in egg extracts from bacteria-injected females.
(DOCX)

S1 Text Additional experiment showing the presence of transcripts of tenecin-1 in eggs of immune challenged females of *Tenebrio molitor*. (DOCX)

S2 Text Additional experiment searching for the presence of a bacterial infection in ovaries and eggs of *Tenebrio molitor* females. (DOCX)

S3 Text Comparisons of fits of the statistical analyses on specificity of the transgenerational immune response. (DOC)
