# Supplementary material for: Trans-generational Immune Priming Protects the Eggs Only against Gram-Positive Bacteria in the Mealworm Beetle
Source: PLoS Pathog. 2015 Oct 2;11(10):e1005178. doi: 10.1371/journal.ppat.1005178 (PMC4592268; doi:10.1371/journal.ppat.1005178)
Supplement: S1 Table — (DOCX) [file ppat.1005178.s001.docx]

**S1 Table Microorganisms used in this study.**

| Name | Strain | Source | Type |
| --- | --- | --- | --- |
| *Arthrobacter globiformis* | CIP 105365 | Institut Pasteur | Gram positive bacteria, Lys-type peptidoglycan |
| *Bacillus thuringiensis var. alesti* | CIP53.1 | Institut Pasteur | Gram positive bacteria, DAP-type peptidoglycan |
| *Bacillus subtilis* | CIP 52.62 | Institut Pasteur | Gram positive bacteria, DAP-type peptidoglycan |
| *Candida albicans* | ATCC-24433 | LGC Standards | Yeast, b-1,3 glucan |
| *Escherichia coli* | D31 | CGSC | Gram negative bacteria, DAP-type peptidoglycan |
| *Metarhizium anisopliae* | IP 1693.87 | Institut Pasteur | Fungus, b-1,3 glucan |
| *Serratia entomophila* | CIP 102919 | Institut Pasteur | Gram negative bacteria, DAP-type peptidoglycan |
| *Serratia marcescens* | ATCC-14756 | LGC Standards | Gram negative bacteria, DAP-type peptidoglycan |
