# Supplementary material for: Trans-generational Immune Priming Protects the Eggs Only against Gram-Positive Bacteria in the Mealworm Beetle
Source: PLoS Pathog. 2015 Oct 2;11(10):e1005178. doi: 10.1371/journal.ppat.1005178 (PMC4592268; doi:10.1371/journal.ppat.1005178)
Supplement: S1 Fig — Treatments: PBS = sham-injected females, Ag = A. globiformis, Bt = B. thuringiensis, Ec = E. coli. a) hemolymph tested on A. globiformis, b) hemolymph tested on E. coli. The left panel (a) shows the anti-A. globiformis activity and the right panel (b) shows the anti-E. coli activity in the hemolymph of female beetles according to their immune treatment. Sample size of each treatment is stated under the female treatment (n = x). Females were injected using the same procedure as described in the Materials and Method section. Six μL of hemolymph per female was collected and diluted in 24 μL of PBS supplemented with PTU. For each female, 3 replicates of 2 μL were tested using inhibition zone assay, as described in the Materials and Method section. The edges of the rectangles represent the first and the third quartiles, the central features are the medians, the dashed lines are the maxima and minima. (DOCX) [file ppat.1005178.s002.docx]

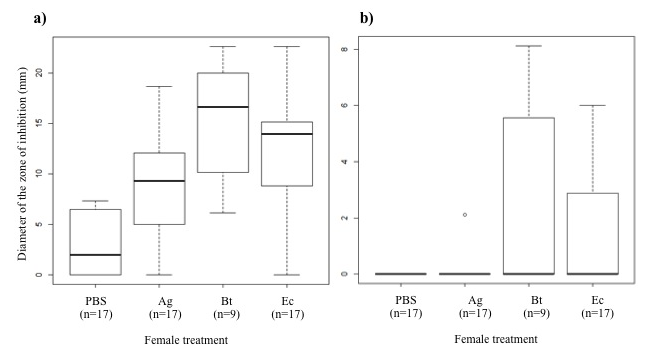


**S1 Fig. Boxplot showing the diameter of the zone of inhibitions (in mm) obtained using hemolymph of females according to their immune treatment.** Treatments: PBS = sham-injected females, Ag = A*. globiformis*, Bt = *B. thuringiensis*, Ec = *E. coli*. The left panel (a) shows the anti-*A. globiformis* activity and the right panel (b) shows the anti-*E. coli* activity in the hemolymph of female beetles according to their immune treatment. Sample size of each treatment is stated under the female treatment (n=x). Females were injected using the same procedure as described in the Materials and Method section. Six µL of hemolymph per female was collected and diluted in 24 µL of PBS supplemented with PTU. For each female, 3 replicates of 2 µL were tested using inhibition zone assay, as described in the Materials and Method section. The edges of the rectangles represent the first and the third quartiles, the central features are the medians, the dashed lines are the maxima and minima.
