# Supplementary figures and images for: Trans-generational Immune Priming Protects the Eggs Only against Gram-Positive Bacteria in the Mealworm Beetle
Source: PLoS Pathog. 2015 Oct 2;11(10):e1005178. doi: 10.1371/journal.ppat.1005178 (PMC4592268; doi:10.1371/journal.ppat.1005178)

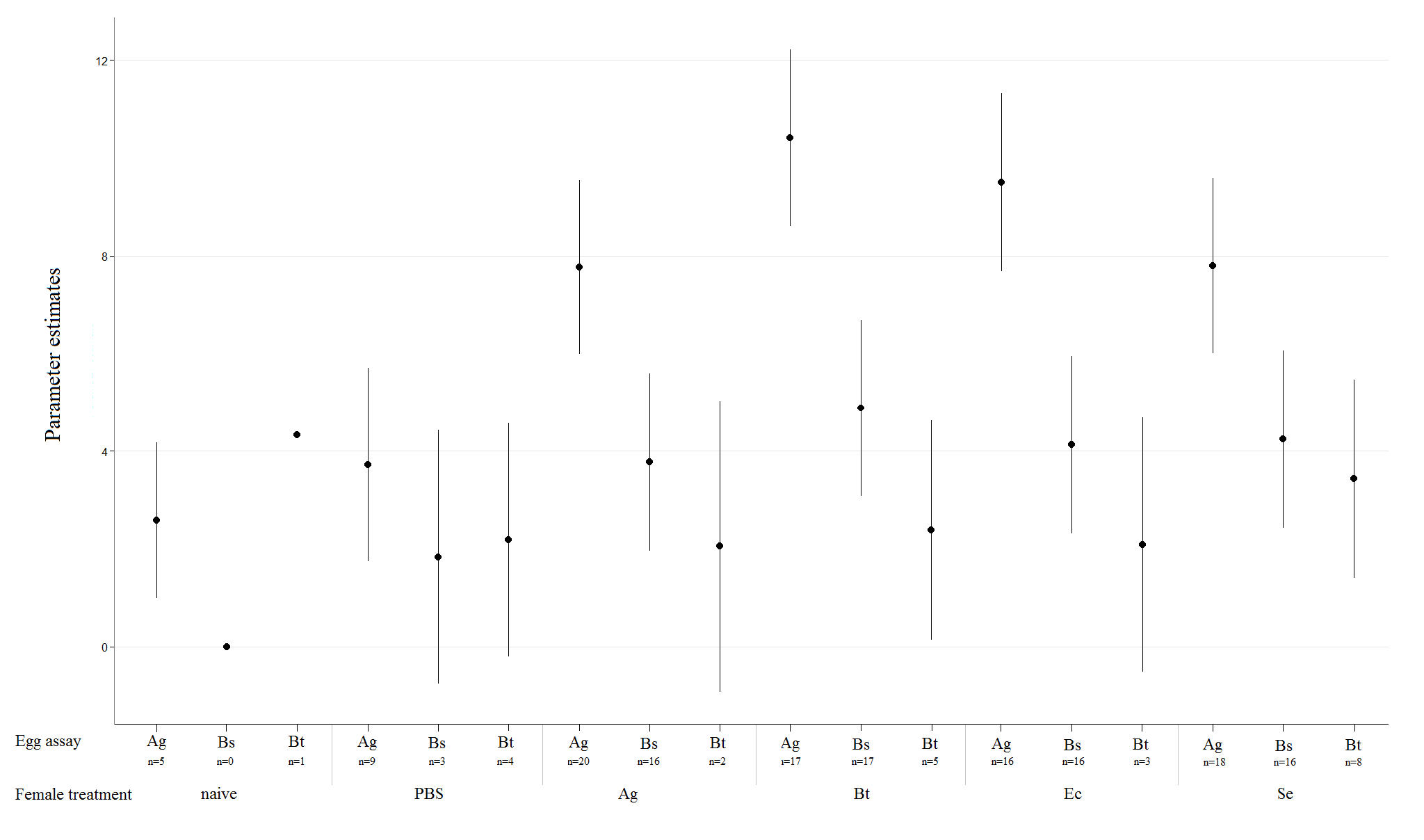

Supplement: S2 Fig — The sample size of each treatment is stated under the egg assay (n = x). Treatments: naïve = unmanipulated females, PBS = Sham injected females, Ag = A. globiformis, Bs = B. subtilis, Bt = B. thuringiensis, Ec = E. coli, Se = S. entomophila. (TIF) [file ppat.1005178.s003.tif]

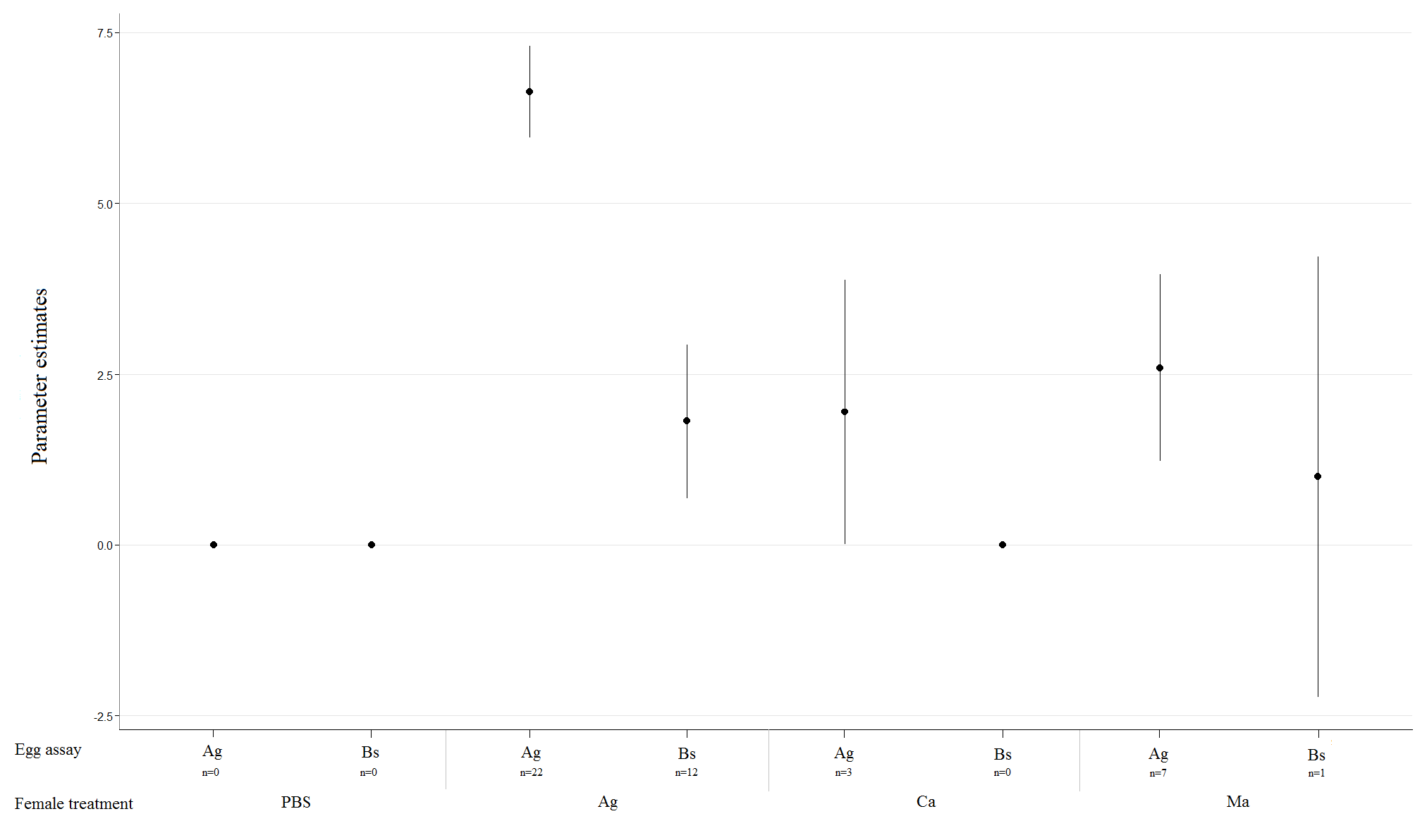

Supplement: S3 Fig — The sample size of each treatment is stated under the egg assay (n = x). Treatments: PBS = Sham injected females, Ag = A. globiformis, Bs = B. subtilis, Ca = C. albicans, Ma = M. anisopliae. (TIF) [file ppat.1005178.s004.tif]
