# Supplementary material for: Trans-generational Immune Priming Protects the Eggs Only against Gram-Positive Bacteria in the Mealworm Beetle
Source: PLoS Pathog. 2015 Oct 2;11(10):e1005178. doi: 10.1371/journal.ppat.1005178 (PMC4592268; doi:10.1371/journal.ppat.1005178)
Supplement: S4 Fig — Antibacterial activity tested against Arthrobacter globiformis of egg extracts from control (PBS, right column) and immune-challenged females with Bacillus thuringiensis (Bt, left column) either non-treated (Bef.), incubated with proteinase K for 2 hours at 37°C (PK) or incubated without proteinase K for 2 hours at 37°C (S/S). Treatment with proteinase K inhibited the antibacterial activity of the egg extract from Bt-immune-challenged females revealing the proteinaceous nature of the antimicrobial compounds in these egg extracts. (DOCX) [file ppat.1005178.s005.docx]

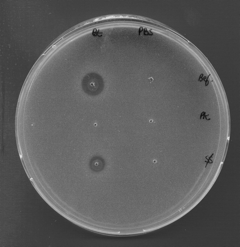


**S4 Fig. Effect of proteinase K treatment on antibacterial activity of egg extracts from immune-challenged females of *Tenebrio molitor*.** Antibacterial activity of egg extracts from control (PBS, right column) and immune-challenged females (injected with inactivated *Bacillus thuringiensis*, Bt, left column) was tested on a Petri dish inoculated with *Arthrobacter globiformis.* Egg extracts were either not treated (Bef.), incubated with proteinase K for 2 hours at 37°C (PK) or incubated without proteinase K for 2 hours at 37°C (S/S). Treatment with proteinase K inhibited the antibacterial activity of the egg extract from Bt-immune-challenged females, revealing the proteinaceous nature of the antimicrobial compounds in these egg extracts.
