# Supplementary material for: Trans-generational Immune Priming Protects the Eggs Only against Gram-Positive Bacteria in the Mealworm Beetle
Source: PLoS Pathog. 2015 Oct 2;11(10):e1005178. doi: 10.1371/journal.ppat.1005178 (PMC4592268; doi:10.1371/journal.ppat.1005178)
Supplement: S5 Fig — Protein profiles of eggs from females injected either with PBS or with B. thuringiensis (Bt) are compared using a Tricine-SDS PAGE gel. The D1 band, only detected in egg extracts from Bt-injected females, is shown in the rectangle. In total, we detected the D1 band in 11 of the 14 protein profiles of egg extracts from Bt-injected females, but never in the 5 egg extracts from sham-injected control females. (DOCX) [file ppat.1005178.s006.docx]

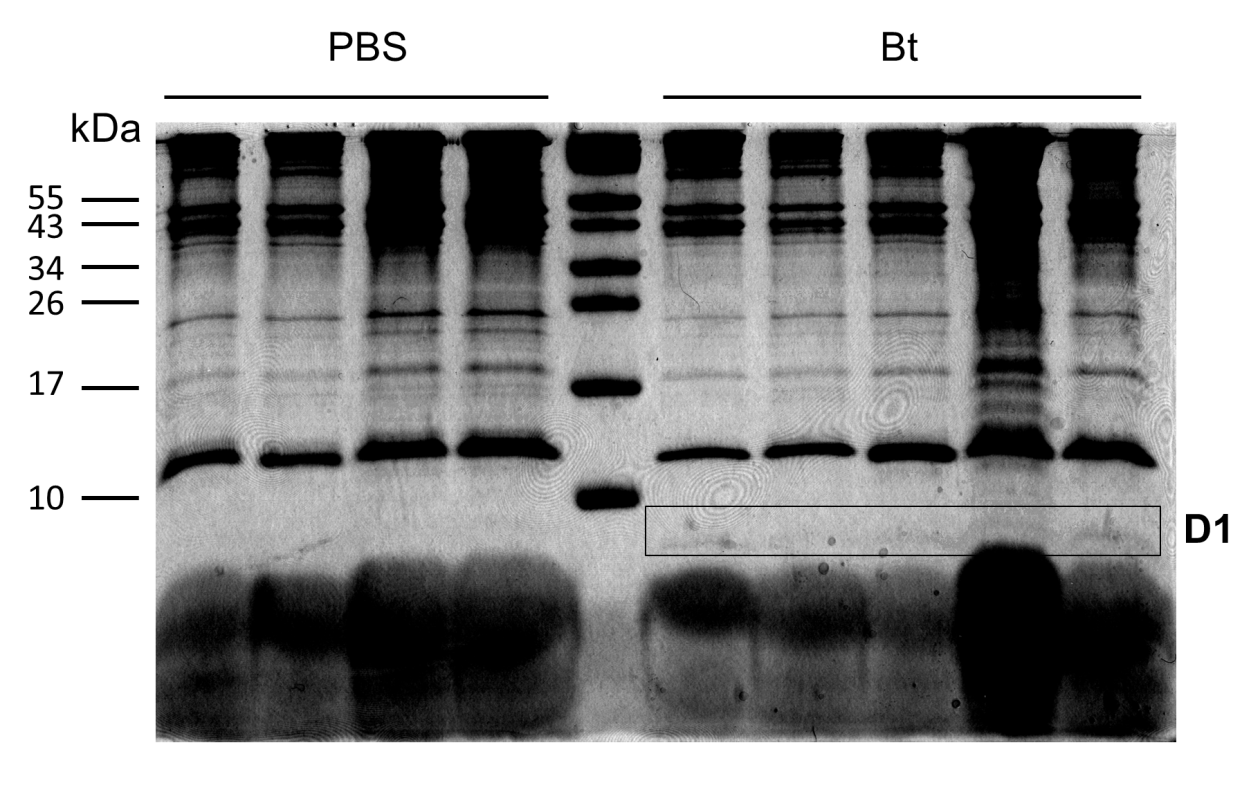


**S5 Fig. Detection of the D1 band in egg extracts from females injected with bacteria.** Protein profiles of eggs from females injected either with PBS or with *B. thuringiensis* (Bt) are compared using a Tricine-SDS PAGE gel. The D1 band, only detected in egg extracts from Bt-injected females, is shown in the rectangle. . In total, we detected the D1 band in 11 of the 14 protein profiles of egg extracts from Bt-injected females, but never in the 5 egg extracts from sham-injected control females.
