# Supplementary material for: Trans-generational Immune Priming Protects the Eggs Only against Gram-Positive Bacteria in the Mealworm Beetle
Source: PLoS Pathog. 2015 Oct 2;11(10):e1005178. doi: 10.1371/journal.ppat.1005178 (PMC4592268; doi:10.1371/journal.ppat.1005178)
Supplement: S6 Fig — Egg extracts from PBS-injected and S. entomophila-injected females were run in duplicate sets on a single native AU-PAGE gel. One half of the gel was stained with colloidal blue (left), the other was used on a Gel Overlay Assay with A. globiformis (right). A zone of bacterial growth inhibition (arrowhead) is observed at the level of the N1 band (arrow), only in egg extracts from bacteria-injected females. (DOCX) [file ppat.1005178.s007.docx]

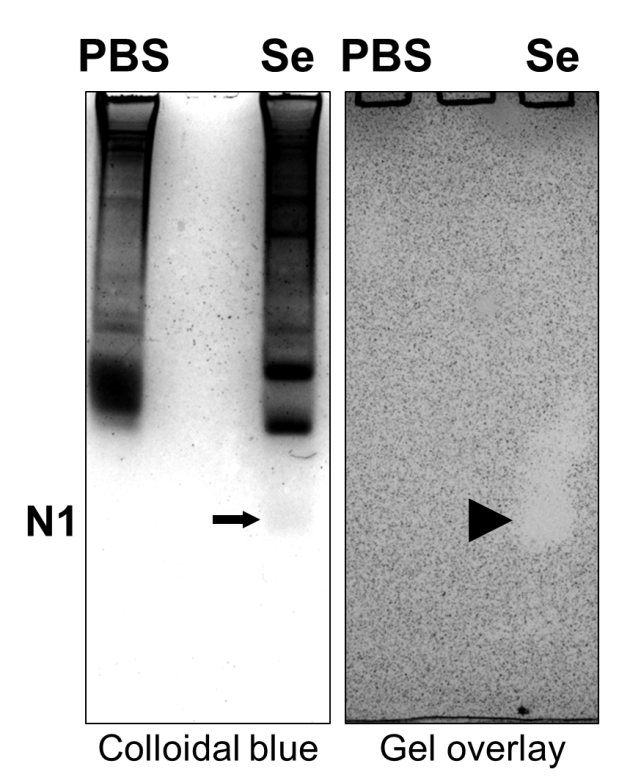


**S6 Fig. Localization of the antimicrobial proteins by Gel Overlay Assay.** Egg extracts from PBS-injected and *S.entomophila*-injected females were run in duplicate sets on a single native AU-PAGE gel. One half of the gel was stained with colloidal blue (left), the other was used on a Gel Overlay Assay with *A. globiformis* (right). A zone of bacterial growth inhibition (arrowhead) is observed at the level of the N1 band (arrow), only in egg extracts from bacteria-injected females.
