# Supplementary material for: Trans-generational Immune Priming Protects the Eggs Only against Gram-Positive Bacteria in the Mealworm Beetle
Source: PLoS Pathog. 2015 Oct 2;11(10):e1005178. doi: 10.1371/journal.ppat.1005178 (PMC4592268; doi:10.1371/journal.ppat.1005178)
Supplement: S1 Text — (DOCX) [file ppat.1005178.s008.docx]

**S1 Text. Supporting Online Material for:**

**Trans-Generational Immune Priming Protects the Eggs only against Gram-positive Bacteria in the Mealworm Beetle**

Aurore Dubuffet¤a, Caroline Zanchi¤b, Gwendoline Boutet, Jérôme Moreau, Maria Teixeira, Yannick Moret

Équipe Écologie Évolutive, UMR CNRS 6282 BioGéoSciences, Université de Bourgogne, Dijon, France.

* Email: yannick.moret@u-bourgogne.fr (YM)

¤a Current address: Laboratoire Microorganismes : Génome et Environnement, UMR CNRS 6023, Université Blaise Pascal, Clermont-Ferrand, France.

¤b Current address: Institute for Biology, Freie Universität Berlin, Berlin, Germany.

**Summary**

This experiment aimed to determine whether the Tenecin-1 found in eggs of *T. molitor* females challenged with inactivated bacteria was provided by the females or produced by the eggs themselves. Under the first scenario, expression levels of the gene coding for Tenecin-1 should not differ between eggs of naïve females and eggs of challenged females, and should be absent or low, while under the second scenario we should observe higher levels of Tenecin-1 expression in eggs of immune challenged females.

Using semi-quantitative RT-PCR, we compared levels of expression of Tenecin-1 in eggs of naïve and immune challenged females. We found that this gene can be expressed in eggs, and that this expression was higher in eggs laid by immune challenged females.

This result suggests that TGIP is more likely explained by a maternal transfer of mRNAs coding for Tenecin-1 or by transgenerational epigenetic changes which induce an increased expression of Tenecin-1 in eggs, rather than by a simple maternal transfer of antimicrobial peptides.

**Materials and Methods**

***Maternal immune challenge and egg collection***

Four 10 days old (± 2 days) adult females of *Tenebrio molitor* were immune challenged by injection of a 5-µL suspension of inactivated *Bacillus thuringiensis* in PBS after being chilled on ice for 10 min. Additional four females were chilled on ice for 10 min but not injected (naïve females). Immediately after manipulation, the females were paired for 2 days with a virgin and immunologically naïve male of the same age in a Petri dish supplied with wheat flour, apple and water in standard laboratory conditions (25°C, 70% RH; dark). Eggs laid from day 2 to day 4 post-challenge were collected for each female.

***RNA extraction & Reverse Transcription***

Immediately after egg collection, 5 eggs per female were crushed into 50 μl of sterile water supplemented with Phenylthiourea acid (PTU) to inhibit melanization. 750 μl of Tri-Reagent (Molecular Research Center) and 20 μl acetic acid 5N were added to each sample. After homogenization, samples were left 5 min at room temperature for lysis, and kept at
-80°C for one week. RNA extraction was then performed according to manufacturer instructions (Molecular Research Center) and RNA extracts were kept at -80°C overnight, after we estimated their concentrations using a Nanodrop. To produce cDNA from the isolated RNA, a reverse transcription was done using the iScript^TM^ cDNA synthesis kit (Biorad) according to manufacturer instructions. Using about 1 μg RNA for each reaction, we eventually obtained similar cDNA concentrations (between 1.12 and 1.20 mg/mL). The obtained cDNA were kept at -80°C.

***Semi quantitative RT-PCR***

Expression levels of Tenecin-1 were compared between eggs of naïve and immune challenged females using in parallel primer pairs for Tenecin-1 (sense, 5’ -ATG AAG CTT ACA ATC TTC GCA- 3’; antisense, 5’-TTA TCT GCA AAC GCA GAC CC-3’), and the control ribosomal protein L-27A (RPL27A) (sense, 5’-GCA TGG CAA ACA CAG AAA GCA TC-3’; antisense, 5’-ATG ACA GGT TGG TTA GGC AGG C-3’) on the same cDNA templates [1]. PCR ampliﬁcations of the target cDNA were performed in a reaction mixture containing 1.25 μl of cDNA, 2.5 μl of PCR HotMaster Taq Buffer (5 Prime), 5 nmoles of each speciﬁc primer, and sterile H_2_0 to make up 25 μl. The following conditions were used: an initial denaturation step at 94°C (2 min), followed by several cycles of 94°C (20 s), 63°C (20 sec), 65°C (20 sec), and a final elongation step of 5 min at 65°C. PCR products obtained from different cycles (i.e. 24, 26 and 28 for RPL27A and 31, 32, 33 and 34 for Tenecin-1) were taken for comparison of the amounts of these products by electrophoresis on 2.3% agarose gels.

**Results**

We obtained similar amounts of PCR products using the internal control primers RPL27A. Tenecin-1 transcripts were detected in 3 out of 4 samples obtained from eggs of naïve females, and in 4 out 4 samples from eggs of Bt-injected females. However, PCR products for this gene were more important using cDNA from eggs of Bt-injected females than with those of naïve females (Figure 1).


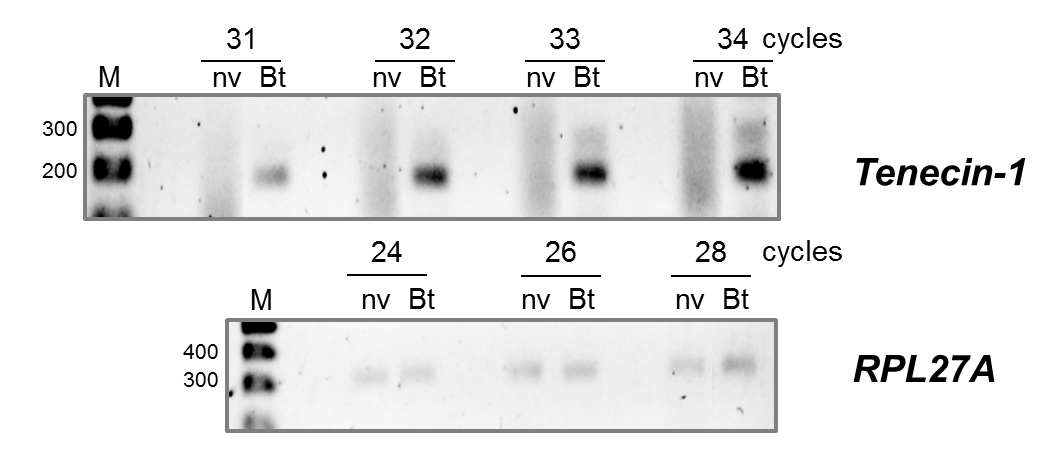


**Figure 1**: Semi-quantitative RT-PCR detecting the expression of Tenecin-1 in *Tenebrio molitor* eggs according to the maternal treatment. M: DNA marker. nv: control females; Bt: females challenged with inactivated *Bacillus thuringiensis*.

**References**

1. Roh K-B, Kim C-H, Lee H, Kwon H-M, Park J-W, Ryu J-W, Kurokawa K, Ha N-C, Lee W-J, Lemaitre B, Söderhäll K, Lee B-L (2009) Proteolytic Cascade for the Activation of the Insect Toll Pathway Induced by the Fungal Cell Wall Component. *J Biol Chem* 284: 19474-19481.
