# Supplementary material for: Trans-generational Immune Priming Protects the Eggs Only against Gram-Positive Bacteria in the Mealworm Beetle
Source: PLoS Pathog. 2015 Oct 2;11(10):e1005178. doi: 10.1371/journal.ppat.1005178 (PMC4592268; doi:10.1371/journal.ppat.1005178)
Supplement: S2 Text — (DOCX) [file ppat.1005178.s009.docx]

**S2 Text. Supporting Online Material for:**

**Trans-Generational Immune Priming Protects the Eggs only against Gram-positive Bacteria in the Mealworm Beetle**

Aurore Dubuffet¤a, Caroline Zanchi¤b, Gwendoline Boutet, Jérôme Moreau, Maria Teixeira, Yannick Moret

Équipe Écologie Évolutive, UMR CNRS 6282 BioGéoSciences, Université de Bourgogne, Dijon, France.

* Email: yannick.moret@u-bourgogne.fr (YM)

¤a Current address: Laboratoire Microorganismes : Génome et Environnement, UMR CNRS 6023, Université Blaise Pascal, Clermont-Ferrand, France.

¤b Current address: Institute for Biology, Freie Universität Berlin, Berlin, Germany.

**Summary**

This supplementary experiment aimed at determining whether antibacterial activity against Gram-positive bacteria found in eggs of immune challenged females of *Tenebrio molitor*, whatever the bacterial strain that challenges mothers, results from the presence of Gram-positive symbionts in ovaries and eggs. Here, it is hypothesized that *T. molitor* houses symbiotic Gram-positive bacteria persisting at an undetectable concentration to the host immune system. Upon infection by another bacterial species, the symbionts would replicate before vertical transmission, which would stimulate the eggs and/or ovaries immune response, thus explaining the presence of antibacterial activity directed toward Gram-positive bacteria in eggs of immune challenge females.

To test for this hypothesis, we searched for the presence of microbial DNA in ovaries and eggs of immune challenged and control females using a PCR assay. Ovaries of few control and immune challenged females presented a weak positive amplification. The analysis of the sequences of these PCR products revealed that they belong to Gram-negative bacteria (e.g. *Serratia sp.* and *Enterobacter sp*.) that may have contaminated the samples either during dissection or/and PCR mix preparation. Overall, these results suggest that *T. molitor* does not harbor bacterial symbionts in ovaries and that antimicrobial activity in eggs of immune challenged females is unlikely to result from a “microbiota effect”.

**Materials and Methods**

Ten adult females of the mealworm, *Tenebrio molitor*, of 10 days old (± 2 days) were immune challenged by injection of a 5-µL suspension of inactivated *Escherichia coli* in phosphate buffer saline (PBS 10mM, pH 7.4) after being chilled on ice for 10 min. Ten control females were treated in the same way, but with the omission of bacteria as a procedural control for effect of the injection (sham control mothers). Immediately after their immune treatment, the females were paired for 3 days with a virgin and immunologically naïve male of the same age in a Petri dish supplied with wheat flour, apple and water in standard laboratory conditions (25°C, 70% RH; dark). Females were then cooled on ice before dissection of their ovaries.

***Dissections***

Dissections were performed in as much clean conditions as possible to avoid contaminations by exogenous microbes. Each female was bathed into sterile water containing 20% of ethanol before dissection and ovary extraction with sterile forceps. Entire ovaries were removed, rinsed in sterile water and immediately collected into a centrifuge tube containing 200 µL of Queen’s lysis buffer and 5 µL of proteinase K (20 mg/mL).

***DNA extraction and PCR***

Ovary samples were incubated in the lysis buffer with of proteinase K overnight before purification of the genomic DNA using a phenol-chloroform method [1]. DNA from cultured *Bacillus thuringiensis* was also extracted as positive control for subsequent PCR amplifications.

The presence of bacterial DNA was assayed using a “universal” set of primers amplifying a fragment of the mitochondrial 16S rDNA gene of bacteria: 27F (5’-AGAGTTTGATCMTGGCTCAG-3’) and 1492R (5’-CGGTTACCTTGTTACGACTT-3’) [2]. DNA from *T. molitor* was assayed using primers that amplify a gene fragment of the cytochrome *c* oxidase subunit I (COI): C1-J-2195 (5′-TTGATTTTTTGGTCATCCAGAAGT-3′) and L2-N-3014 (5′-TCCAATGCACTAATCTGCCATATTA-3′) [3, 4]. The amplification of the COI gene fragment was used to control the integrity of the DNA samples.

PCR assays were performed using 2 µL of each DNA template in a total reaction volume of 20 µL. The PCR reaction mix contained 0.2 µM of dinucleotide triphosphates (dNTPs), 2.5 mM of Mg^2+^, 0.5 units of HotMaster^TM^ *Taq* DNA polymerase (5PRIME) and 0.2 µM of each primer. PCR conditions were an initial denaturation step of 2 min at 94 °C, followed by 20 s at 94°C (denaturation), 20 s at 54°C (primer annealing) and 1 min 30 s at 65°C (primer extension) for 35 cycles, and a 5 min final extension at 65°C. PCR products were separated on 1.5% agarose gels and visualized by ethidium bromide staining and a UV light source.

***Sequencing, sequence editing and alignment***

Successfully amplified PCR products for the mitochondrial 16S rRNA gene were purified and thereafter the sequencing reactions were performed by an external company (Macrogen Europe). All fragments were sequenced in forward direction using the 27F primer. Sequences generated were identified against the GenBank database on NCBI (<http://blast.ncbi.nlm.nih.gov/Blast.cgi>) using Molecular Evolutionary Genetics Analysis (MEGA 6) software [5, 6].

**Results**

The COI gene fragment from *T. molitor* was successfully amplified for all the samples, confirming DNA integrity. Amplification of the 16S rDNA gene gave a fragment of approximately 1450 bp with DNA from *Bacillus thurigiensis*. In *T. molitor*, the amplification gave a very slight band with DNA samples for 7 samples out of 20 (3 from control and 4 from challenged females). Despite the weak signal we were able to sequence these fragments in two samples. Both were identified as Gram-negative bacteria: one was identified as belonging to the *Serratia* genus (100% identity with several *Serratia* species, based on 1447 bp), the other to the *Enterobacter* genus (99% identity with several *Enterobacter* species, based on 1447 bp).

**References**

1. Hillis DM, Mable BK, Larson A, Davis SK, Zimmer EA. (1996) Nucleic acids IV: sequencing and cloning. In Hillis DM, Moritz C, Mable BK, editors. Molecular Systematics, Sinnauer Associates, Sunderland, MA.
2. Weisburg WG, Barns SM, Pelletier DA, Lane DJ (1991) 16S ribosomal DNA amplification for phylogenetic study. J Bacteriol 173:697-703.
3. Simon C, Frati F, Beckenbach A, Crespi B, Liu H, Flook P (1994) Evolution, weighting, and phylogenetic utility of mitochondrial gene sequences and a compilation of conserved polymerase chain reaction primers. Ann Entomol Soc Am 87: 651–702.
4. Frohlich DR, Torres-Jerez I, Bedford ID, Markham PG, Brown JK (1999) A phylogeographical analysis of the *Bemisia tabaci* species complex based on mitochondrial DNA markers. Mol Ecol 8: 1683–1691.
5. Altschul SF, Gish W, Miller W, Myers EW, Lipman DJ (1990) Basic local alignment search tool. J Mol Biol 215:403-410.
6. Tamura K, Stecher G, Peterson D, Filipski A, Kumar S (2013) MEGA6: Molecular Evolutionary Genetics Analysis Version 6.0. Mol Biol Evol 30: 2725-2729.
