# Supplementary material for: Trans-generational Immune Priming Protects the Eggs Only against Gram-Positive Bacteria in the Mealworm Beetle
Source: PLoS Pathog. 2015 Oct 2;11(10):e1005178. doi: 10.1371/journal.ppat.1005178 (PMC4592268; doi:10.1371/journal.ppat.1005178)
Supplement: S3 Text — (DOC) [file ppat.1005178.s010.doc]

**S3 Text. Supporting Online Material for:**

**Trans-Generational Immune Priming Protects the Eggs only against Gram-positive Bacteria in the Mealworm Beetle**

Aurore Dubuffet¤a, Caroline Zanchi¤b, Gwendoline Boutet, Jérôme Moreau, Maria Teixeira, Yannick Moret

Équipe Écologie Évolutive, UMR CNRS 6282 BioGéoSciences, Université de Bourgogne, Dijon, France.

* Email: yannick.moret@u-bourgogne.fr (YM)

¤a Current address: Laboratoire Microorganismes : Génome et Environnement, UMR CNRS 6023, Université Blaise Pascal, Clermont-Ferrand, France.

¤b Current address: Institute for Biology, Freie Universität Berlin, Berlin, Germany.

This appendix provides details on the fits of the statistical models presented in the text of the manuscript.

| **Model** | **Degrees of freedom** | **Residual deviance** |
| --- | --- | --- |
| Null | 0, 532 | 676.19 |
| Female treatment * egg assay | 20, 503 | 238.51 |
| Female treatment + egg assay | 9, 523 | 266.04 |
| Female treatment | 4, 528 | 598.04 |
| Egg assay | 5, 527 | 396.51 |

**Table 1** Comparison of the fits of the Generalized Linear Models (GLM) testing the proportion of egg clutches showing a zone of inhibition according to the female treatment (naive, PBS-injected, or challenged with *Arthrobacter globiformis*, *Bacillus thuringiensis*, *Escherischia coli* or *Serratia entomophila*) and the egg assay (*A. globiformis*, *Bacillus subtilis*, *B. thuringiensis*, *E. coli* or *Serratia marcescens*).

| **Model** | **Degrees of freedom** | **Residual deviance** |
| --- | --- | --- |
| Null | 0, 175 | 3.18 |
| Female treatment * egg assay | 16, 159 | 1.8 |
| Female treatment + egg assay | 7, 168 | 1.97 |
| Female treatment | 5, 170 | 3 |
| Egg assay | 2, 173 | 2.4 |

**Table 2** Comparison of the fits of the Linear Models (LM) carried on protected egg extracts, testing the size of the zone of inhibition according to the female treatment (naive, PBS-injected, or challenged with *A. globiformis*, *B. thuringiensis*, *E. coli* or *S. entomophila*) and the egg assay (*A. globiformis*, *B. subtilis*, *B. thuringiensis*).

| **Model** | **Degrees of freedom** | **Residual deviance** |
| --- | --- | --- |
| Null | 0, 365 | 272.86 |
| Female treatment * egg assay | 28, 348 | 91.17 |
| Female treatment + egg assay | 10, 358 | 91.82 |
| Female treatment | 7, 363 | 241.15 |
| Egg assay | 3, 360 | 147.62 |

**Table 3** Comparisons of the fits of the Generalized Linear Models (GLM) testing the proportion of egg clutches showing a zone of inhibition according to the female treatment (PBS-injected, or challenged with *A. globiformis*, *Candida albicans* or *Metarhizium anisopliae*) and the egg assay (*A. globiformis*, *B. subtilis*, *E. coli*, *S. marcescens*, *C. albicans* or *M. anisopliae*).

| **Model** | **Degrees of freedom** | **Residual deviance** |
| --- | --- | --- |
| Null | 0, 44 | 2.78 |
| Female treatment * egg assay | 4, 40 | 1.56 |
| Female treatment + egg assay | 3, 41 | 1.6 |
| Female treatment | 2, 42 | 2.58 |
| Egg assay | 1, 43 | 2.27 |

**Table 4** Comparison of the fits of the Linear Models (LM) carried on egg clutches showing a zone of inhibition, testing the size of the zone of inhibition according to the female treatment (PBS-injected, or challenged with *A. globiformis*, *C. albicans* or *M. anisopliae*) and the egg assay (*A. globiformis*, *B. subtilis*).
